# Unique Information and Secret Key Agreement

**DOI:** 10.3390/e21010012

**Published:** 2018-12-24

**Authors:** Ryan G. James, Jeffrey Emenheiser, James P. Crutchfield

**Affiliations:** Complexity Sciences Center and Physics Department, University of California at Davis, One Shields Avenue, Davis, CA 95616, USA

**Keywords:** information theory, partial information decomposition, secret key agreement, cryptography

## Abstract

The partial information decomposition (PID) is a promising framework for decomposing a joint random variable into the amount of influence each source variable $X_{i}$ has on a target variable *Y*, relative to the other sources. For two sources, influence breaks down into the information that both $X_{0}$ and $X_{1}$ redundantly share with *Y*, what $X_{0}$ uniquely shares with *Y*, what $X_{1}$ uniquely shares with *Y*, and finally what $X_{0}$ and $X_{1}$ synergistically share with *Y*. Unfortunately, considerable disagreement has arisen as to how these four components should be quantified. Drawing from cryptography, we consider the secret key agreement rate as an operational method of quantifying unique information. Secret key agreement rate comes in several forms, depending upon which parties are permitted to communicate. We demonstrate that three of these four forms are inconsistent with the PID. The remaining form implies certain interpretations as to the PID’s meaning—interpretations not present in PID’s definition but that, we argue, need to be explicit. Specifically, the use of a consistent PID quantified using a secret key agreement rate naturally induces a directional interpretation of the PID. We further reveal a surprising connection between third-order connected information, two-way secret key agreement rate, and synergy. We also consider difficulties which arise with a popular PID measure in light of the results here as well as from a maximum entropy viewpoint. We close by reviewing the challenges facing the PID.

## 1. Introduction

Consider a joint distribution over “source” variables $X_{0}$ and $X_{1}$ and “target” *Y*. Such distributions arise in many settings: sensory integration, logical computing, neural coding, functional network inference, and many others. One promising approach to understanding how the information shared between $\left(X_{0},X_{1}\right)$, and *Y* is organized is the partial information decomposition (PID) [1]. This decomposition seeks to quantify how much of the information shared between $X_{0}$, $X_{1}$, and *Y* is done so redundantly, how much is uniquely attributable to $X_{0}$, how much is uniquely attributable to $X_{1}$, and finally how much arises synergistically by considering both $X_{0}$ and $X_{1}$ together.

Unfortunately, the lack of a commonly accepted method of quantifying these components has hindered PID’s adoption. In point of fact, several proposed axioms are not mutually consistent [2,3]. Furthermore, to date, there is little agreement as to which should hold. Here, we take a step toward understanding these issues by adopting an operational definition for the unique information. This operational definition comes from information-theoretic cryptography and quantifies the rate at which two parties can construct a secret while a third party eavesdrops. Said more simply, for a source and the target to uniquely share information, no other variables can have any portion of that information—their uniquely shared information is a secret that only the source and target have.

There are four varieties of secret key agreement rate depending on which parties are allowed to communicate, each of which defines a different PID. Each variety also relates to a different intuition as to how the PID operates. We discuss several aspects of these different methods and further demonstrate that three of the four fail to construct an internally consistent decomposition. The surviving method induces a directionality on the PID that has not been explicitly considered before.

Our development proceeds as follows. Section 2 briefly describes the two-source PID. Section 3 reviews the notion of secret key agreement rate and how to quantify it in three contexts: no one communicates, only Alice communicates, and both Alice and Bob communicate. Section 4 discusses the behavior of the PID quantified utilizing secret key agreement rates as unique information and what intuitions are implied by the choice of who is permitted to communicate. Section 5 compares the behavior of the one consistent secret key agreement rate PID with several others proposed in the literature. Section 6 explores two further implications of our primary results, first in a distribution where two-way communication seems to capture synergistic, third-order connected information and second in the behavior of an extant method of quantifying the PID along with maximum entropy methods. Finally, Section 7 summarizes our findings and speculates about PID’s future.

## 2. Partial Information Decomposition

Two-source PID seeks to decompose the mutual information I[$X_{0}$$X_{1}$ : *Y*] between “sources” $X_{0}$ and $X_{1}$ and a “target” *Y* into four nonnegative components. The components identify information that is redundant, uniquely associated with $X_{0}$, uniquely associated with $X_{1}$, and synergistic:

(1) $$\begin{array}{ccc} I[X_{0}X_{1}:Y]= & \;I_{\partial }\left[X_{0}\cdot X_{1}\to Y\right] & \;\mathrm{redundant}\\ & +I_{\partial }\left[X_{0}\to Y\backslash X_{1}\right] & \;\mathrm{unique}\mathrm{with}X_{0}\\ & +I_{\partial }\left[X_{1}\to Y\backslash X_{0}\right] & \;\mathrm{unique}\mathrm{with}X_{1}\\ & +I_{\partial }\left[X_{0}X_{1}\to Y\right]\; & \;\mathrm{synergistic}. \end{array}$$

Furthermore, the mutual information I[$X_{0}:Y$] between $X_{0}$ and *Y* is decomposed into two components:

(2) $$\begin{array}{ccc} I[X_{0}:Y]= & \;I_{\partial }\left[X_{0}\cdot X_{1}\to Y\right] & \;\mathrm{redundant}\\ & +I_{\partial }\left[X_{0}\to Y\backslash X_{1}\right]\; & \;\mathrm{unique}\mathrm{with}X_{0} \end{array}$$

and, similarly:

(3) $$\begin{array}{ccc} I[X_{1}:Y]= & \;I_{\partial }\left[X_{0}\cdot X_{1}\to Y\right] & \;\mathrm{redundant}\\ & +I_{\partial }\left[X_{1}\to Y\backslash X_{0}\right]\; & \;\mathrm{unique}\mathrm{with}X_{1}. \end{array}$$

In this way, PID relates the four component pieces of information. However, since Equations (1) to (3) provide only three independent constraints for four quantities; it does not uniquely determine how to quantify them in general. That is, this fourth constraint lies outside of the PID.

By the same logic, though, the decomposition is uniquely determined by quantifying exactly one of its constituents. In the case that one wishes to directly quantify the unique information $I_{\partial }\left[X_{0}\to Y\backslash X_{1}\right]$ and $I_{\partial }\left[X_{1}\to Y\backslash X_{0}\right]$ , a consistency relation must hold so that they do not overconstrain the decomposition:

(4) $$\begin{array}{c} I_{\partial }\left[X_{0}\to Y\backslash X_{1}\right]+\left[X_{1}:Y\right]=I_{\partial }\left[X_{1}\to Y\backslash X_{0}\right]+\left[X_{0}:Y\right].\; \end{array}$$

This ensures that using either Equation (2) or Equation (3) results in the same quantification of $I_{\partial }\left[X_{0}\cdot X_{1}\to Y\right]$ .

## 3. Secret Key Agreement

Secret key agreement is a fundamental concept within information-theoretic cryptography [4]. Consider three parties—Alice, Bob, and Eve—who each partially observe a source of common randomness, joint probability distribution A,B,E∼p(a,b,e), where Alice has access only to *a*, Bob *b*, and Eve *e*. The central challenge is to determine if it is possible for Alice and Bob to agree on a secret key of which Eve has no knowledge. The degree to which they may generate such a secret key immediately depends on the structure of the joint distribution A,B,E. It also depends on whether Alice and Bob are allowed to publicly communicate.

Concretely, consider Alice, Bob, and Eve each receiving *n* independent, identically distributed samples according to p(a,b,e)—Alice receiving $A^{n}$, Bob $B^{n}$, and Eve $E^{n}$, where $X^{n}$ denotes a sequence of random variables $X_{1},X_{2},\ldots ,X_{n}$. Note that, although each party’s observations $X_{i}$, $X_{j}$ are independent, the observations of different parties at the same time are correlated according to p(a,b,e). A secret key agreement scheme consists of functions *f* and *g*, as well as a protocol *h* for public communication allowing either Alice, Bob, neither, or both to communicate. In the case of a single party being permitted to communicate—say, Alice—she constructs $C=h(A^{n})$ and then broadcasts it to all parties over an authenticated channel. In the case that both parties are permitted communication, they take turns constructing and broadcasting messages of the form $C_{i}=h_{i}\left(A^{n},C_{\left[0,\ldots ,i-1\right]}\right)$ (Alice) and $C_{i}=h_{i}\left(B^{n},C_{\left[0,\ldots ,i-1\right]}\right)$ (Bob) [5]. Said more plainly, Alice’s public messages are a function of her observations and any prior public communication from both parties. Bob’s public messages are a function of his observations and any prior communication from both parties.

Formally, a secret key agreement scheme is considered *R*-achievable if for all ϵ>0:

$$\begin{array}{cc} K_{A} & \overset{\left(1\right)}{=}f\left(A^{n},C\right)\;,\\ K_{B} & \overset{\left(2\right)}{=}g\left(B^{n},C\right)\;,\\ p(K_{A}=K_{B}=K) & \overset{\left(3\right)}{\geq }1-\epsilon \;,\\ I[K:C,E^{n}] & \overset{\left(4\right)}{\leq }\epsilon \;,\;\mathrm{and}\\ \frac{1}{n}H[K] & \overset{\left(5\right)}{\geq }R-\epsilon \;, \end{array}$$

where (1) and (2) denote the method by which Alice and Bob construct their keys $K_{A}$ and $K_{B}$, respectively, (3) states that their keys must agree with arbitrarily high probability, (4) states that the information about the key which Eve—armed with both her private information $E^{n}$ as well as the public communication *C*—has access is arbitrarily small, and (5) states that the key consists of approximately *R* bits per sample.

The greatest rate *R* such that an achievable scheme exists is known as the secret key agreement rate. Notational variations indicate which parties are permitted to communicate. In the case that Alice and Bob are not allowed to communicate, their rate of secret key agreement is denoted S(A:B||E). When only Alice is allowed to communicate, their secret key agreement rate is S(A→B||E) or, equivalently, S(B←A||E). When both Alice and Bob are allowed to communicate, their secret key agreement rate is denoted S(A↔B||E). In this, we modified the standard notation for secret key agreement rates to emphasize which party or parties communicate.

The secret key agreement rates obey a simple partial order. S(A:B||E) lower bounds both S(A→B||E) and S(A←B||E), since no communication is a special case of one party communicating. Similarly, both S(A→B||E) and S(A←B||E) lower bound S(A↔B||E), since only one party communicating is a special case of both parties communicating. Other than having some identical lower and upper bounds, S(A→B||E) and S(A←B||E) are themselves generally incomparable.

In the case of no communication, S(A:B||E) is given by [6]:

(5) $$\begin{array}{c} S(A:B\;||\;E)=H[A⋏B|E],\; \end{array}$$

where X⋏Y denotes the Gács–Körner common random variable [7]. It is worth noting that the entropy of this variable, the Gács–Körner common information, is not continuous under smooth changes in the probability distribution. It is also only nonzero for a measure-zero set of distributions within the simplex; specifically, the set of distributions whose joint events form bipartite graphs with multiple connected components [8].

In the case of one-way communication, S(A→B||E) is given by [9]:

(6) $$\begin{array}{c} S\left(A\to B\;||\;E\right)=\max\left\{B:K|C-E:K|C\right\},\; \end{array}$$

where the maximum is taken over all variables *C* and *K*, such that the following Markov condition holds: C⊸−K⊸−A⊸−BE. This quantifies the maximum amount of information that Bob can share with the key above the amount that Eve shares with the key, both given the public communication. It suffices, by the Fenchel–Eggleston strengthening of Carathéodory’s theorem [10], to assume *K* and *C* alphabets are limited: |K|≤|A| and $\left|C\right|\leq {\left|A\right|}^{2}$.

There is no such closed-form, calculable solution for S(A↔B||E); however, various upper- and lower-bounds are known [5]. One simple lower bound is the supremum of the two one-way secret key agreement rates, as they are both a subset of bidirectional communication. An even simpler upper bound that we will use is the intrinsic mutual information [11]:

(7) $$\begin{array}{c} I[A:B↓E]=\min_{p(\bar{e}|e)}I[A:B|\bar{E}].\; \end{array}$$

This states that the amount of secret information that Alice and Bob share is no greater than their mutual information conditioned on any modification of Eve’s observations. Here, $\bar{E}$ is an arbitrary stochastic function of *E* or alternatively the result of passing *E* through a memoryless channel.

The unique PID component $I_{\partial }\left[X_{0}\to Y\backslash X_{1}\right]$ could be assigned the value of a secret key agreement rate under four different schemes. First, neither $X_{0}$ nor *Y* may be allowed to communicate. Second, only $X_{0}$ can communicate. Third, only *Y* is permitted to communicate. Finally, both $X_{0}$ and *Y* may be allowed to communicate. Note that the eavesdropper $X_{1}$ is not allowed to communicate in any secret sharing schemes here.

Secret key agreement rates have been associated with unique information before. One particular upper bound on S(A↔B||E)—the intrinsic mutual information Equation (7)—is known to not satisfy the consistency condition Equation (4) [12]. Rosas et al. [13] briefly explored the idea of eavesdroppers influence on the PID. More recently, the relationship between a particular method of quantifying unique information and one-way secret key agreement $S(X_{0}\leftarrow Y\;||\;X_{1})$ has been considered [14]. Furthermore, there are analogous notions of secret key agreement rates within the channel setting, as opposed to the source setting considered here. We leave an analysis of that setting as future work.

## 4. Cryptographic Partial Information Decompositions

**Figure 1:**
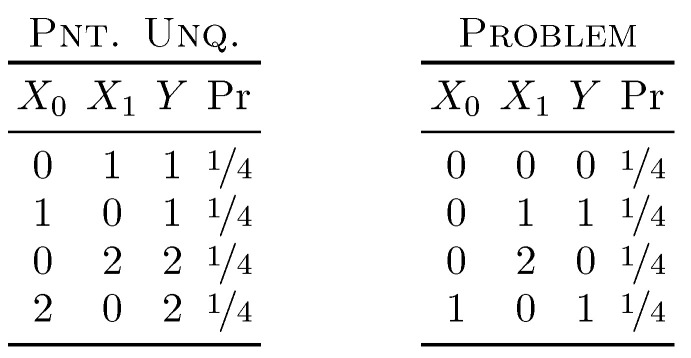
Two distributions of interest: The first, Pointwise Unique, exemplifies the directionality inherent in the one-way secret key agreement rates. The second, Problem, demonstrates that the no-communication, one-way communication with the source communicating (“camel”), and the two-way communication secret key agreement rates result in inconsistent PIDs (partial information decomposition).

We now address the application of each form of secret key agreement rate as unique information in turn. For each resulting PID, we consider two distributions. The first is that called Pointwise Unique, chosen here to exemplify the differing intuitions that can be applied to the PID. The second distribution we look at is entitled Problem as it serves as a counterexample demonstrating that three of the four forms of secret key agreement do not result in a consistent decomposition. Both distributions are given in Figure 1. Although we only consider two distributions here, their behaviors are rich enough for us to draw out the two main results of this work. Further examples are given in Section 5.

Interpreting the Pointwise Unique [15] distribution is relatively straightforward. The target *Y* takes on the values “1” and “2” with equal probability. At the same time, exactly one of the two sources (again with equal probability) will be equal to *Y*, while the other is “0”. The mutual information I$\left[X_{0}:Y\right]=½$ bit and I$\left[X_{1}:Y\right]=½$ bit.

The Problem distribution lacks the symmetry of Pointwise Unique, yet still consists of four equally probable events. The sources are restricted to take on pairs “00”, “01”, “02”, “10”. The target *Y* is equal to a “1” if either $X_{0}$ or $X_{1}$ is “1”, and is “0” otherwise. With this distribution, the mutual information I$[X_{0}:Y]=0.3113\;\mathrm{bit}$ and I$[X_{1}:Y]=½$ bit.

### 4.1. No Public Communication

In the first case, we consider the unique information from $X_{i}$ to *Y* as the rate at which $X_{i}$ and *Y* can agree on a secret key while exchanging no public communication: $I_{\partial }\left[X_{i}\to Y\backslash X_{j}\right]\;=\;S(X_{i}:Y\;||\;X_{j})$. This approach has some appeal: the PID is defined simply by a joint distribution without any express allowance or prohibition on public communication. However, given its quantification in terms of the Gács-Körner common information, the quantity $S(X_{i}:Y\;||\;X_{j})$ does not vary continuously with the distribution of interest. Now, what is the behavior of this measure on our two distributions of interest?

When applied to Pointwise Unique, each source and the target are unable to construct a secret key. In turn, each unique information is determined to be 0 bits. This results in a redundancy and a synergy each of ½ bit.

The Problem distribution demonstrates the inability of $S(X_{i}:Y\;||\;X_{j})$ to construct a consistent PID. In this instance, as in the case of Pointwise Unique, no secrecy is possible and each unique information is assigned a value of 0 bits. We therefore determine from Equation (2) that the redundancy should be I$\left[X_{0}:Y\right]-I_{\partial }\left[X_{0}\to Y\backslash X_{1}\right]=0.3113\;\mathrm{bit}-0\;\mathrm{bit}=0.3113\;\mathrm{bit}$. Equation (3), however, says that the redundancy is I$[X_{1}:Y]-I_{\partial }\left[X_{1}\to Y\backslash X_{0}\right]=1/2\;\mathrm{bit}-0\;\mathrm{bit}=1/2\;\mathrm{bit}$. This contradiction demonstrates that no-communication secret key agreement rate cannot be used as a PID’s unique  components.

The resulting partial information decompositions for both distributions are listed in Table 1.

### 4.2. One-Way Public Communication

We next consider the situation when one of the two parties is allowed public communication. This gives us two options: either the source $X_{i}$ communicates to target *Y* or vice versa. Both situations enshrine a particular directionality in the resulting PID.

The first, where $X_{i}$ constructs $C=h(X_{i}^{n})$ and publicly communicates it, emphasizes the channels $X_{i}\to Y$ and the channel $X_{0}X_{1}\to Y$. This creates a narrative of the sources conspiring to create the target. We call this interpretation the camel intuition, after the aphorism that a camel is a horse designed by committee. The committee member $X_{i}$ may announce what design constraints they brought to the table.

The second option, where *Y* constructs $C=h(Y^{n})$ and publicly communicates it, emphasizes the channels $Y\to X_{i}$ and $Y\to X_{0}X_{1}$. It implies the situation that the sources are imperfect representations of the target. We call this interpretation the elephant intuition, as it recalls the parable of the blind men describing an elephant for the first time. The elephant *Y* may announce which of its features is revealed in a particular instance.

#### 4.2.1. Camels

The first option adopts $I_{\partial }\left[X_{i}\to Y\backslash X_{j}\right]=S(X_{i}\to Y\;||\;X_{j})$, bringing to mind the idea of sources acting as inputs into some scheme by which the target is produced. When viewed this way, one may ask questions such as “How much information in $X_{0}$ is uniquely conveyed to *Y*?”. Furthermore, the channels $X_{0}\to Y$, $X_{1}\to Y$, and $X_{0}X_{1}\to Y$ take center stage.

Through this lens, the Pointwise Unique distribution has a clear interpretation. Given any realization, exactly one source is perfectly correlated with the target, while the other is impotently “0”. From this vantage, it is clear that the unique information should each be ½ bit, and this is borne out with the one-way secret key agreement rate. To see this, consider $X_{0}$ broadcasting each time they observed a “1” or a “2”. This corresponds to the joint events “101” and “202”, respectively. It is clear that, when considering just these two events, $X_{0}$ and *Y* can safely utilize their observations which will agree exactly and with which $X_{1}$ has no knowledge. Since these events occur half the time, we conclude that the secret key agreement rate is ½ bit. This implies that the redundancy and synergy of this decomposition are both 0 bits.

For the Problem distribution, we find that $X_{1}$ can broadcast the times when they observed a “1” or a “2”, which correspond to *Y* having observed a “1” or “0”, respectively. In both instances, $X_{0}$ observed a “0” and so cannot deduce what the other two have agreed upon. This leads to $S(X_{1}\to Y\;||\;X_{0})$ being equal to ½ bit. At the same time, $S(X_{0}\to Y\;||\;X_{1})$ vanishes. However, Problem’s redundancy and synergy cannot be quantified, since the two secret key agreement schemes imply different redundancies and so are inconsistent with Equation (4).

The resulting PIDs for both are given in Table 2.

#### 4.2.2. Elephants

When the target *Y* is the one party permitted communication, one adopts $I_{\partial }\left[X_{i}\to Y\backslash X_{j}\right]=S(X_{i}\leftarrow Y\;||\;X_{j})$ and we can interpret the sources as alternate views of the singular target. Consider, for example, journalism where several sources give differing perspectives on the same event. When viewed this way, one might ask a question such as “How much information in *Y* is uniquely captured by $X_{0}$?”. The channels $X_{0}\leftarrow Y$, $X_{1}\leftarrow Y$, and $X_{0}X_{1}\leftarrow Y$ are paramount with this approach. We denote these in reverse to emphasize that *Y* is still the *target* in the PID.

Considered this way, the Pointwise Unique distribution takes on a different character. The sources each receive identical descriptions of the target—accurate half the time and erased the remainder. The description is identical, however. Nothing is uniquely provided to either source. This is reflected in the secret key agreement rates: *Y* can broadcast her observation, restricting events to either “011” and “101” or to “022” and “202”. In either case, these restrictions do not help in the construction of a secret key since *Y* cannot further restrict to cases where it is $X_{0}$ that agrees with her and not $X_{1}$. This makes each unique information 0 bits, leaving both the redundancy and synergy ½ bit.

The Problem distribution’s unique information are $S(X_{0}\leftarrow Y\;||\;X_{1})=0$ bit and $S(X_{1}\leftarrow Y\;||\;X_{0})=0.1887$ bit. Unlike the prior two decompositions, this unique information satisfies Equation (4). The resulting redundancy is 0.3113 bit while the synergy is ½ bit.

Their PIDs are listed in Table 3. Thus, by having *Y* publicly communicate and thus invoking a particular directionality, we finally get a consistent PID.

### 4.3. Two-Way Public Communication

We finally turn to the full two-way secret key agreement rate: $I_{\partial }\left[X_{i}\to Y\backslash X_{j}\right]=S(X_{i}↔Y\;||\;X_{j})$. This approach is also appealing, as it does not ascribe any directionality to interpreting the PID. Furthermore, it varies continuously with the distribution, unlike the no-communication case. However, this quantity is generally impossible to compute directly, with only upper and lower bounds known. Fortunately, this only slightly complicates the analyses we wish to make.

In the case of the Pointwise Unique distribution, it is not possible to extract more secret information than was done in the camel situation. Therefore, the resulting PID is identical: unique information of ½ bit and redundancy and synergy of 0 bits.

Problem, however, is again a problem. In this instance, upper and lower bounds on $S(X_{1}↔Y\;||\;X_{0})$ converge: the larger of the two one-way secret key agreement rates form a lower bound of ½ bit, while the upper bound provided by the intrinsic mutual information is also ½ bit, and so we know this value exactly. Utilizing the consistency relation Equation (4), we find that the other unique information must be 0.3113 bit in order for the full decomposition to be consistent. However, the intrinsic mutual information places an upper bound of 0.1887 bit on $S(X_{0}↔Y\;||\;X_{1})$. We therefore must conclude that two-way secret key agreement rates cannot be used to directly quantify unique information and a consistent PID cannot be built using them.

The resulting PIDs for both these distributions can be seen in Table 4.

### 4.4. Summary

To conclude, then, there is only one secret-key communication scenario—*Y* publicly communicates—that yields a consistent PID, as in Table 3. The above arguments by counterexample pruned away the unworkable scenarios, narrowing to only one. Naturally, this does not constitute proof that the remaining scenario always leads to a consistent PID. The narrowing, though, allowed us to turn to numerical searches using the dit [16] software package. Extensive searches were unable to find a counterexample. Thus, practically, with a high probability, this scenario leads to consistent PIDs.

Though this is the singular viable secret key agreement rate-based PID, we hesitate to fully endorse its use due to the necessary directionality that comes with it. That is, one must invoke a directionality, unspecified by the PID, to have a consistent PID when using secret key agreement as the basis for the PID component of unique information. It is not immediately obvious as to why only $S(X_{i}\leftarrow Y\;||\;X_{j})$ results in a viable partial information decomposition, if this is indeed the case. We leave a proof as to whether $I_{\partial }\left[X_{0}\to Y\backslash X_{1}\right]=S(X_{0}\leftarrow Y\;||\;X_{1})$ and $I_{\partial }\left[X_{1}\to Y\backslash X_{0}\right]=S(X_{1}\leftarrow Y\;||\;X_{0})$ satisfy Equation (4) for all distributions or not as an open question. Specifically:

(8) $$\begin{array}{c} \max_{K,C}I[X_{0}:K|C]-I[X_{1}:K|C]+I[X_{1}:Y]\overset{?}{=}\max_{K,C}I[X_{1}:K|C]-I[X_{0}:K|C]+I[X_{0}:Y], \end{array}$$

where both optimizations are performed over the space $X_{0}X_{1}⊸\;-Y⊸\;-K⊸\;-C$. We conjecture that $S(X_{i}\leftarrow Y\;||\;X_{j})$ is the only secret key agreement rate resulting in a viable PID due to the fact that the spaces in which the solutions are found are identical.

The reasons why the other three secret key agreement rates fail to form consistent decompositions are likely particular to each scenario. In the case of no communication, the limitations carried over from the Gács–Körner common information play a major role—specifically, that it vanishes even for weakly mixing distributions. In the case of the one-way “camel” secret key agreement rate, it is possible that the failure arises from the optimization spaces of each unique information being different. Finally, for the case of two-way communication, we offer several speculations in Section 6.1.

These measures of unique information can also be applied within the multivariate sources setting [1], though, like other measures of unique information [17,18], these measures cannot fully quantify the general decompositions there.

## 5. Examples

**Figure 2:**
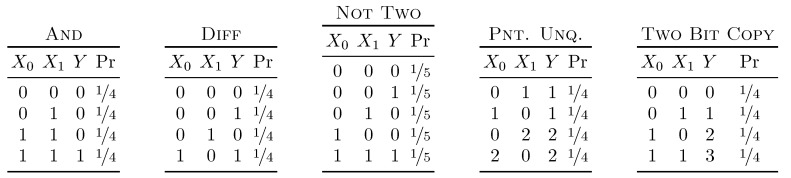
The five distributions we utilize to compare partial information decompositions.

We next demonstrate the behavior of the partial information decomposition quantified using $I_{\partial }\left[X_{i}\to Y\backslash X_{j}\right]=S(X_{i}\leftarrow Y\;||\;X_{j})$, herein denoted $I_{\leftarrow }$. On many of the “standard” distributions—Rdn, Unq, Xor— $I_{\leftarrow }$ behaves as intuited by Griffith [19]. Here, we compare its behavior with that of several other proposed measures— $I_{\min}$ [1], $I_{\mathrm{proj}}$ [20], $I_{\mathrm{BROJA}}$ [17], $I_{\mathrm{CCS}}$ [21], $I_{\mathrm{dep}}$ [18], $I_{\pm }$ [15], and $I_{\mathrm{RR}}$ [22]—across five distributions—And, Diff, Not Two, Pnt. Unq., and Two Bit Copy. These distributions are given in Figure 2. Note that Reference [14] proved that the secret key agreement rates $S(X_{i}\leftarrow Y\;||\;X_{j})$ lower bound the unique information of $I_{\mathrm{BROJA}}$.

The And distribution—the first set of results in Table 5—yields the same decomposition under $I_{\min}$, $I_{\mathrm{proj}}$, $I_{\mathrm{BROJA}}$, and $I_{\leftarrow }$. In each of these cases, there is no unique information, resulting in 0.311 bit of redundancy and ½ bit of synergy. $I_{\pm }$ produces negative unique values of −¼ bit with a redundancy of 0.561 bit and a synergy of ¾ bit. $I_{\mathrm{CCS}}$, $I_{\mathrm{dep}}$, and $I_{\mathrm{RR}}$ all produce positive unique values, indicating that, at least in this case, they interpret unique information as something more than the ability of source and target to agree on a secret when the target can communicate.

The Diff distribution—the second set of results in Table 5—is named so due to its keen ability to differentiate PID measures. In this example, two pairs of PIDs coincide: $I_{\min}$ with $I_{\mathrm{CCS}}$ and $I_{\mathrm{BROJA}}$ with $I_{\mathrm{dep}}$. In magnitude, $I_{\leftarrow }$ attributes the least to unique information for this distribution, while both $I_{\mathrm{BROJA}}$ and $I_{\mathrm{dep}}$ attribute the most.

The Not Two distribution—the third set of results in Table 5—is named due to it consisting of all binary events over three variables that do not contain two “1”s. As far as the behavior of the various PIDs goes, the distribution is very similar to the And distribution. $I_{\min}$, $I_{\mathrm{proj}}$, $I_{\mathrm{BROJA}}$, and $I_{\leftarrow }$ all allot 0 bit to the unique information, while $I_{\pm }$ finds the unique information to be negative. Here, $I_{\mathrm{CCS}}$ also finds them to be negative. There is a major difference between the Not Two and And distributions, however: the Not Two possesses a great deal of third-order information, while the And possesses none. This indicates that, although the PIDs of the two distributions are qualitatively similar, their structures are in fact quite distinct.

Next, we consider the Pnt. Unq. distribution—the fourth set of results in Table 5—again. We see that $I_{\min}$, $I_{\mathrm{proj}}$, $I_{\mathrm{BROJA}}$, and $I_{\leftarrow }$ behave as elephants, while $I_{\mathrm{CCS}}$ and $I_{\pm }$ behave as camels. $I_{\mathrm{RR}}$ does not commit to either but leans towards elephant, while $I_{\mathrm{dep}}$ splits the difference.

Finally, we consider the venerable Two Bit Copy distribution, and the final set of results in Table 5. Here, $I_{\min}$ and $I_{\pm }$ stand out as assigning one bit to redundant information and one bit to synergy, while assigning nothing to unique. All other measures assign one bit to each unique piece of information and nothing to redundancy and synergy. Note that this is not a directionality issue: all four secret key agreement rates agree that the rate at which a secret key can be constructed is 1 bit.

## 6. Discussion

We now turn to two follow-on developments arising from the tools developed thus far. First, we define a distribution whose two-way secret key agreement rates behave in a curious manner with very interesting implications regarding the nature of information itself. Second, we take a closer look at an alternative proposal for quantifying unique information and describe its behavior in relationship to the camel-elephant dichotomy defined in Section 4.

### 6.1. When Conversation Is More Powerful Than a Lecture

**Figure 3:**
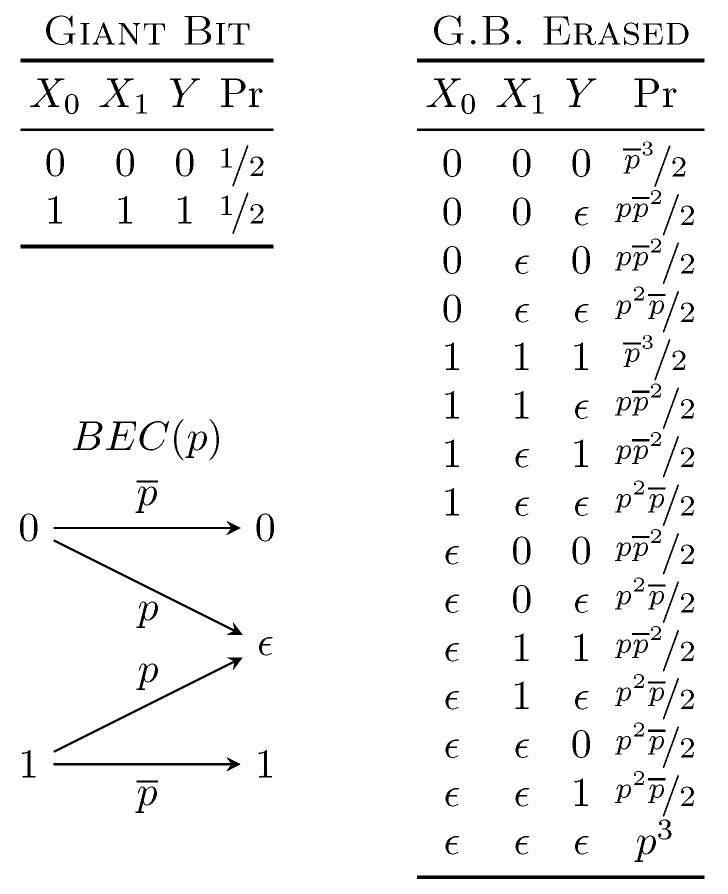
Distribution whose one-way secret key agreement rates are all 0 bits, yet has a nonzero two-way secret key agreement rate. It is constructed from the Giant Bit distribution by passing each variable independently through a binary erasure channel BEC(p) with erasure probability *p*. This distribution has a two-way secret key agreement rate of $p{\bar{p}}^{2}$ between any two variables with the third as an eavesdropper.

We now explore the PID quantified by two-way secret key agreement further. Though this does not generally form a consistent decomposition, in our exploration of its behavior, we discovered an interesting phenomena independent of its use as a measure of unique information. Consider the Giant Bit distribution, which exemplifies redundant information. The distribution G.B. Erased, resulting from passing each variable through an independent binary erasure channel (BEC), exhibits many interesting properties. It is listed in Figure 3. Most notably, the one-way secret key agreement rates between any two variables with the third eavesdropping vanish. However, the two-way secret key agreement rate is equal to $p{\bar{p}}^{2}=I[X_{i}:Y|X_{j}]$ [23]. Furthermore, notice that subtracting Equation (3) from Equation (1) tells us that:

(9) $$\begin{array}{cc} I[X_{0}X_{1}:Y]-I[X_{1}:Y] & =I[X_{0}:Y|X_{1}]\\ & =I_{\partial }\left[X_{0}\to Y\backslash X_{1}\right]+I_{\partial }\left[X_{0}X_{1}\to Y\right]. \end{array}$$

That is, the conditional mutual information is equal to unique information plus synergistic information.

Evaluating the PID using $S(X_{i}↔Y\;||\;X_{j})$ as unique information results, in this case, in a consistent decomposition. Furthermore, the redundant and synergistic information are zero. This is, however, troublesome: G.B. Erased possesses nonzero third-order connected information [24], a quantity commonly considered a component of the synergy [18]. Indeed, it is provably attributed to synergy by both the $I_{\mathrm{dep}}$ [18] and $I_{\mathrm{BROJA}}$ [17] methods, and likely others as well. No other proposed method of quantifying the PID results in zero redundancy or synergy. The implication here is that, if indeed the third-order connected information is a form of synergy, the two-way secret key agreement rate overestimates unique information by including some types of synergistic effect. Therefore, we conclude that bidirectional communication between two parties can, in some instances, determine information held solely in trivariate interactions. This may underlie the inability of a two-way secret key agreement rate to form a consistent partial information decomposition: in some instances, the third-order connected information is attributed to both unique information when it should be attributed solely to the synergistic information. It remains to understand (i) how independently and identically transforming a distribution with no third-order connected information can result in its creation and (ii) how only two of the variables can recover it when allowed to communicate.

### 6.2. $I_{\mathrm{BROJA}}$, the Elephant

The measure $I_{\mathrm{BROJA}}$ of Bertschinger et al. [17] is perhaps the most widely accepted and used method of quantifying the PID. Though popular, it has its detractors [15,21]. Here, we interpret the criticisms leveled and $I_{\mathrm{BROJA}}$ as a product of camel intuitions being applied to an elephantesque [14] measure. In doing so, we will primarily consider the Pointwise Unique distribution.

As noted in Section 4.2, if a source is permitted to communicate with the target, then a secret key agreement rate of ½ bit is achievable, while, if the target communicates with the source, then it is impossible to agree on a secret key. From this camel perspective, it is clear that each source uniquely determines the target, half the time. The elephant perspective, however, allots nothing to unique information as each source is provided with identical information. This would greatly disconcert the camel and may lead one to think that the elephant has “artificially inflated” the redundancy [21]. We next take a closer look at this notion, using $I_{\mathrm{BROJA}}$.

**Figure 4:**
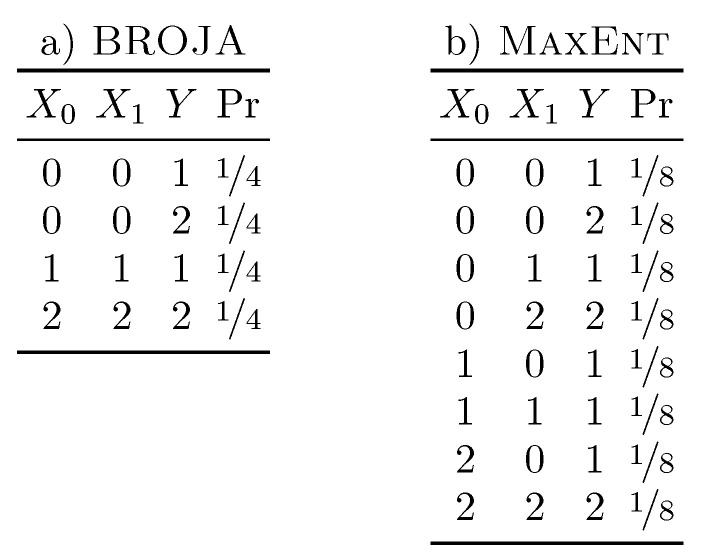
Two modified forms of the Pointwise Unique distribution. (**a**) intermediate distribution resulting from the BROJA optimization. It has the minimum sources-target mutual information consistent with the source-target marginals; (**b**) maximum entropy distribution consistent with the source-target marginals. It contains no structure beyond that implied by those marginals.

In the course of computing $I_{\mathrm{BROJA}}$ for the distribution $p(X_{0},X_{1},Y)$, the set of distributions:

$$\begin{array}{c} Q=\left\{q\left(X_{0},X_{1},Y\right):\forall i,\;q\left(X_{i},Y\right)=p\left(X_{i},Y\right)\right\} \end{array}$$

is considered. The (⁎) assumption [17] is then invoked, which states that redundancy and all unique information are constant within this family of distributions. To complete the quantification, the distribution with minimum I[$X_{0}$$X_{1}$ : *Y*] is selected from this family. The resulting distribution associated with the Pointwise Unique distribution can be seen in Figure 4. Made explicit, it can now be seen that $I_{\mathrm{BROJA}}$ does indeed correlate the sources, but, under assumption (⁎), this does not affect the redundancy.

One aspect of $I_{\mathrm{BROJA}}$ and assumption (⁎) we believe warrants further investigation is its relationship with maximum entropy philosophy [25]. The latter is, in effect, Occam’s razor applied to probability distributions: given a set of constraints, the most natural distribution to associate with them is that with maximum entropy. As it turns out, this is equivalent to the distribution nearest the unstructured product-of-marginals distribution $\bar{p}\left(x,y,z,\ldots \right)=p\left(x\right)p\left(y\right)p\left(z\right)\ldots$ [26]:

$$\begin{array}{c} \underset{q\in Q}{\arg\max}H[q]=\underset{q\in Q}{\arg\max}D_{\mathrm{KL}}[q\;||\;\bar{p}]\;, \end{array}$$

where $D_{\mathrm{KL}}[P\;||\;P]$ is the relative entropy between distributions *P* and *Q*. Having briefly introduced the ideas behind maximum entropy, we next cast their light on the BROJA optimization employed to calculate $I_{\mathrm{BROJA}}$.

Let us first consider the distribution resulting from BROJA optimization. Its entropy is unchanged from the Pointwise Unique distribution indicating that it has the same amount of structure—they are equally distant from the product distribution. The BROJA distribution has reduced I[$X_{0}$$X_{1}$ : *Y*] mutual information, however, indicating perhaps that the optimization has shifted some of the distribution’s structure away from the sources–target interaction. It is interesting that this optimization could not simply remove the synergy from the distribution altogether, resulting in a larger entropy.

If one takes assumption (⁎) and directly applies the maximum entropy philosophy, a different distribution results. This distribution, seen in Figure 4, has a larger entropy than both the Pointwise Unique and the BROJA intermediate distribution, indicating that it in fact has less structure than either. Under assumption (⁎), the MaxEnt distribution, also in Figure 4, retains all the redundant and unique information, while under maximum entropy it contains no structure not implied by the source-target marginals—specifically, no synergy. The combination of assumption (⁎) and maximum entropy philosophy does not, however, result in a viable partial information decomposition; the maximization of entropy can result in source–target mutual information which exceeds that of the original distribution.

To be clear, this is not to claim that assumption (⁎) or BROJA optimization are wrong or incorrect, only that the optimization’s behavior in light of well-established maximum entropy principles is subtle and requires careful investigation. For example, it may be that the source-target marginals do imply some level of triadic interaction and therefore the maximum entropy distribution reflects this lingering synergy. At the same time, BROJA minimization may be capable of maintaining that level of structure implied by the marginals, but somehow shunts it into H[*Y*|$X_{0}$$X_{1}$].

## 7. Conclusions

At present, a primary barrier for PID’s general adoption as a useful and possibly central tool in analyzing how complex systems store and process information is an agreement on a method to quantify its component information. Here, we posited that one reason for disagreement stems from conflicting intuitions regarding the decomposition’s operational behavior. To give an operational meaning to unique information and address these intuitions, we equated unique information with the ability of two parties to agree on a secret—a reasonably intuitive operationalization of what it means for two variables to share a piece of information that no others have. This led to numerous observations.

The first is that the PID, as currently defined, is ambivalent to any notion of directionality. There are, however, very clear cases in which the assumption of a directionality—or lack thereof—is critical to the existence of unique information. Consider, for example, the case of the McGurk effect [27] where the visual stimulus of one phoneme and the auditory stimulus of another phoneme gives rise to the perception of a third phoneme. By construction, the stimuli cause the perception, and the channels implicit in a camel intuition are central. If one were to study this interaction using an elephant-like PID, it is unclear that the resulting decomposition would reflect the neurobiological mechanisms by which the perception is produced. Similarly, a camel-like measure would be inappropriate when interpreting simultaneous positron emission tomography (PET) and magnetic resonance imaging (MRI) scans of a tumor.

One can view this as the PID being inherently context-dependent and conclude that quantification requires specifying directionality. In this case, the elephant intuition is apparently more natural, as adopting closely-related notions from cryptography results in a consistent PID. If context demands the camel intuition, though, either a noncryptographic method of quantifying unique information is needed or consistency must be enforced by augmenting the secret key agreement rate. It is additionally possible that associating secret key agreement rates with unique information is fundamentally flawed and that, ultimately, PID entails quantifying unique information as something distinct from the ability to agree on a secret key. Whatever is missing has yet to be identified.

The next observation concerns the third-order connected information. We first demonstrated that such triadic information can be constructed from common information in which each constituent variable is independently and identically modified. Furthermore, it was shown that any two of three parties, when engaging in bidirectional communication, capture the triadic information. Granted, this does not generically occur. For example, if $X_{0}$$X_{1}$*Y* are related by Xor, the distribution contains 1 bit of third-order connected information, but $S(X_{0}↔Y\;||\;X_{1})$ (or any permutation of the variables) is equal to 0 bits. This suggests that the third-order connected information may not be an atomic quantity, but rather consists of two parts, one accessible to two communicating parties and one not. This idea has been explored in a different context in Reference [28].

Our third observation regards the behavior of the $I_{\mathrm{BROJA}}$ measure, especially in relation to standard maximum entropy principles. We first demonstrated that $I_{\mathrm{BROJA}}$ indeed correlates sources, but argued that this behavior only seems inappropriate when adopting a camel intuition. We then discussed how its intermediate distribution is as structured as the initial one and so, if indeed $I_{\mathrm{BROJA}}$ is operating correctly, it must shunt the dependencies that result in synergy to another aspect of the distribution. Finally, we discussed how the standard maximum entropy approach may remove synergy from a distribution all together. This calls for a more careful investigation as to whether it does (and BROJA optimization is incorrect) or does not and if synergistic information arises from source-target marginals and Occam’s razor.

Looking to the future, we trust that this exploration of the relationship between cryptographic secrecy and unique information will provide a basis for future efforts to understand and quantify the partial information decomposition. Furthermore, the explicit recognition of the role that directional intuitions play in the meaning and interpretation of a decomposition should reduce cross-talk and improve understanding as we collectively move forward.   

## Figures and Tables

**Table 1:** Partial information decompositions of Pointwise Unique and Problem when quantified using no-communication secret key agreement rate. Pointwise Unique decomposes into 0 bits for either unique information and into ½ bit for both the redundancy and synergy. Problem’s redundancy and synergy cannot be quantified, since the two secret key agreement rates result in different quantifications. We have denoted this with the symbol **✗**.

$S(X_{i}:Y\;||\;X_{j})$		
Pnt. Unq.	$I_{\partial }\left[X_{0}X_{1}\to Y\right]$	½ bit
	$I_{\partial }\left[X_{0}\to Y\backslash X_{1}\right]$	0 bit
	$I_{\partial }\left[X_{1}\to Y\backslash X_{0}\right]$	0 bit
	$I_{\partial }\left[X_{0}\cdot X_{1}\to Y\right]$	½ bit
Problem	$I_{\partial }\left[X_{0}X_{1}\to Y\right]$	**✗**
	$I_{\partial }\left[X_{0}\to Y\backslash X_{1}\right]$	0 bit
	$I_{\partial }\left[X_{1}\to Y\backslash X_{0}\right]$	0 bit
	$I_{\partial }\left[X_{0}\cdot X_{1}\to Y\right]$	**✗**

**Table 2:** Partial information decompositions of Pointwise Unique and Problem when quantified using one-way communication secret key agreement rate with the source permitted public communication: Pointwise Unique decomposes into ½ bit for either unique information and into 0 bits for both the redundancy and synergy. Problem’s redundancy and synergy cannot be quantified, indicated by an **✗**.

$S(X_{i}\to Y\;||\;X_{j})$		
Pnt. Unq.	$I_{\partial }\left[X_{0}X_{1}\to Y\right]$	0 bits
	$I_{\partial }\left[X_{0}\to Y\backslash X_{1}\right]$	½ bit
	$I_{\partial }\left[X_{1}\to Y\backslash X_{0}\right]$	½ bit
	$I_{\partial }\left[X_{0}\cdot X_{1}\to Y\right]$	0 bits
Problem	$I_{\partial }\left[X_{0}X_{1}\to Y\right]$	**✗**
	$I_{\partial }\left[X_{0}\to Y\backslash X_{1}\right]$	0 bits
	$I_{\partial }\left[X_{1}\to Y\backslash X_{0}\right]$	½ bit
	$I_{\partial }\left[X_{0}\cdot X_{1}\to Y\right]$	**✗**

**Table 3:** PID (partial information decomposition) for Pointwise Unique and Problem when quantified using one-way communication secret key agreement rate with the target permitted public communication: Pointwise Unique decomposes into ½ bit for either unique information, and into 0 bit for both the redundancy and synergy. Problem admits unique information of 0 bit and 0.1887 bit, respectively. This results in a redundancy of 0.3113 bit and a synergy of ½ bit, providing a consistent PID.

$S(X_{i}\leftarrow Y\;||\;X_{j})$		
Pnt. Unq.	$I_{\partial }\left[X_{0}X_{1}\to Y\right]$	½ bit
	$I_{\partial }\left[X_{0}\to Y\backslash X_{1}\right]$	0 bit
	$I_{\partial }\left[X_{1}\to Y\backslash X_{0}\right]$	0 bit
	$I_{\partial }\left[X_{0}\cdot X_{1}\to Y\right]$	½ bit
Problem	$I_{\partial }\left[X_{0}X_{1}\to Y\right]$	½ bit
	$I_{\partial }\left[X_{0}\to Y\backslash X_{1}\right]$	0 bit
	$I_{\partial }\left[X_{1}\to Y\backslash X_{0}\right]$	0.1887 bit
	$I_{\partial }\left[X_{0}\cdot X_{1}\to Y\right]$	0.3113 bit

**Table 4:** PID for Pointwise Unique and Problem when quantified using two-way communication secret key agreement rate: Pointwise Unique decomposes into ½ bit for either unique information, and into 0 bits for both the redundancy and synergy. Problem’s redundancy and synergy cannot be quantified because the two secret key agreement rates result in different quantifications indicated by the symbol **✗**.

$S(X_{i}↔Y\;||\;X_{j})$		
Pnt. Unq.	$I_{\partial }\left[X_{0}X_{1}\to Y\right]$	0 bit
	$I_{\partial }\left[X_{0}\to Y\backslash X_{1}\right]$	½ bit
	$I_{\partial }\left[X_{1}\to Y\backslash X_{0}\right]$	½ bit
	$I_{\partial }\left[X_{0}\cdot X_{1}\to Y\right]$	0 bit
Problem	$I_{\partial }\left[X_{0}X_{1}\to Y\right]$	**✗**
	$I_{\partial }\left[X_{0}\to Y\backslash X_{1}\right]$	≤ 0.1887 bit
	$I_{\partial }\left[X_{1}\to Y\backslash X_{0}\right]$	½ bit
	$I_{\partial }\left[X_{0}\cdot X_{1}\to Y\right]$	**✗**

**Table 5:** Comparison of $I_{\leftarrow }$ with other proposed methods of quantifying the PID.

	$I_{\partial }$	$I_{\min}$	$I_{\mathrm{proj}}$	$I_{\mathrm{BROJA}}$	$I_{\mathrm{ccs}}$	$I_{\mathrm{dep}}$	$I_{\pm }$	$I_{\mathrm{RR}}$	$I_{\leftarrow }$
And	$X_{0}X_{1}$	0.500 bit	0.500 bit	0.500 bit	0.292 bit	0.270 bit	0.750 bit	0.189 bit	0.500 bit
	$X_{0}\backslash X_{1}$	0.000 bit	0.000 bit	0.000 bit	0.208 bit	0.230 bit	-0.250 bit	0.311 bit	0.000 bit
	$X_{1}\backslash X_{0}$	0.000 bit	0.000 bit	0.000 bit	0.208 bit	0.230 bit	-0.250 bit	0.311 bit	0.000 bit
	$X_{0}\cdot X_{1}$	0.311 bit	0.311 bit	0.311 bit	0.104 bit	0.082 bit	0.561 bit	0.000 bit	0.311 bit
Diff	$X_{0}X_{1}$	0.085 bit	0.042 bit	0.000 bit	0.085 bit	0.000 bit	0.292 bit	0.029 bit	0.117 bit
	$X_{0}\backslash X_{1}$	0.104 bit	0.146 bit	0.189 bit	0.104 bit	0.189 bit	-0.104 bit	0.160 bit	0.072 bit
	$X_{1}\backslash X_{0}$	0.104 bit	0.146 bit	0.189 bit	0.104 bit	0.189 bit	-0.104 bit	0.160 bit	0.072 bit
	$X_{0}\cdot X_{1}$	0.208 bit	0.165 bit	0.123 bit	0.208 bit	0.123 bit	0.415 bit	0.151 bit	0.240 bit
Not Two	$X_{0}X_{1}$	0.551 bit	0.551 bit	0.551 bit	0.553 bit	0.536 bit	0.634 bit	0.531 bit	0.551 bit
	$X_{0}\backslash X_{1}$	0.000 bit	0.000 bit	0.000 bit	-0.002 bit	0.015 bit	-0.083 bit	0.020 bit	0.000 bit
	$X_{1}\backslash X_{0}$	0.000 bit	0.000 bit	0.000 bit	-0.002 bit	0.015 bit	-0.083 bit	0.020 bit	0.000 bit
	$X_{0}\cdot X_{1}$	0.020 bit	0.020 bit	0.020 bit	0.022 bit	0.005 bit	0.103 bit	0.001 bit	0.020 bit
Pnt. Unq.	$X_{0}X_{1}$	0.500 bit	0.500 bit	0.500 bit	0.000 bit	0.250 bit	0.000 bit	0.333 bit	0.500 bit
	$X_{0}\backslash X_{1}$	0.000 bit	0.000 bit	0.000 bit	0.500 bit	0.250 bit	0.500 bit	0.167 bit	0.000 bit
	$X_{1}\backslash X_{0}$	0.000 bit	0.000 bit	0.000 bit	0.500 bit	0.250 bit	0.500 bit	0.167 bit	0.000 bit
	$X_{0}\cdot X_{1}$	0.500 bit	0.500 bit	0.500 bit	0.000 bit	0.250 bit	0.000 bit	0.333 bit	0.500 bit
Two Bit	$X_{0}X_{1}$	1.000 bit	0.000 bit	0.000 bit	0.000 bit	0.000 bit	1.000 bit	0.000 bit	0.000 bit
	$X_{0}\backslash X_{1}$	0.000 bit	1.000 bit	1.000 bit	1.000 bit	1.000 bit	0.000 bit	1.000 bit	1.000 bit
	$X_{1}\backslash X_{0}$	0.000 bit	1.000 bit	1.000 bit	1.000 bit	1.000 bit	0.000 bit	1.000 bit	1.000 bit
	$X_{0}\cdot X_{1}$	1.000 bit	0.000 bit	0.000 bit	0.000 bit	0.000 bit	1.000 bit	0.000 bit	0.000 bit

## References

[1] Williams P.L., Beer R.D. (2010). Nonnegative decomposition of multivariate information. arXiv.

[2] Rauh J., Banerjee P., Olbrich E., Jost J., Bertschinger N. (2017). On extractable shared information. Entropy.

[3] Rauh J. (2017). Secret sharing and shared information. Entropy.

[4] Maurer U.M. (1993). Secret key agreement by public discussion from common information. IEEE Trans. Inf. Theory.

[5] Gohari A., Günlü O., Kramer G. (2017). Coding for positive rate in the source model key agreement problem. arXiv.

[6] Chitambar E., Fortescue B., Hsieh M.-H. (2018). The conditional common information in classical and quantum secret key distillation. IEEE Trans. Inf. Theory.

[7] Gács P., Körner J. (1973). Common information is far less than mutual information. Prob. Control Inf. Theory.

[8] Salamatian S., Cohen A., Médard M. (2016). Maximum Entropy Functions: Approximate Gacs-Korner for Distributed Compression. arXiv.

[9] Ahlswede R., Csiszár I. (1993). Common randomness in information theory and cryptography. I. Secret sharing. IEEE Trans. Inf. Theory.

[10] Wyner A., Ziv J. (1976). The rate-distortion function for source coding with side information at the decoder. IEEE Trans. Inf. Theory.

[11] Maurer U.M., Wolf S. (1999). Unconditionally secure key agreement and the intrinsic conditional information. IEEE Trans. Inf. Theory.

[12] Bertschinger N., Rauh J., Olbrich E., Jost J. (2013). Shared information—New insights and problems in decomposing information in complex systems. Proceedings of the European Conference on Complex Systems 2012.

[13] Rosas F., Ntranos V., Ellison C.J., Pollin S., Verhelst M. (2016). Understanding interdependency through complex information sharing. Entropy.

[14] Banerjee P.K., Olbrich E., Jost J., Rauh J. (2018). Unique information and deficiencies. arXiv.

[15] Finn C., Lizier J.T. (2018). Pointwise partial information decomposition using the specificity and ambiguity lattices. Entropy.

[16] James R.G., Ellison C.J., Crutchfield J.P. (2018). Dit: A Python package for discrete information theory. J. Open Source Softw..

[17] Bertschinger N., Rauh J., Olbrich E., Jost J., Ay N. (2014). Quantifying unique information. Entropy.

[18] James R.G., Emenheiser J., Crutchfield J.P. (2019). Unique information via dependency constraints. J. Phys. A.

[19] Griffith V., Koch C. (2014). Quantifying synergistic mutual information. Guided Self-Organization: Inception.

[20] Harder M., Salge C., Polani D. (2013). Bivariate measure of redundant information. Phys. Rev. E.

[21] Ince R.A.A. (2017). Measuring multivariate redundant information with pointwise common change in surprisal. Entropy.

[22] Goodwell A.E., Kumar P. (2017). Temporal information partitioning: Characterizing synergy, uniqueness, and redundancy in interacting environmental variables. Water Resour. Res..

[23] Gohari A., Anantharam V. (2017). Comments on “information-theoretic key agreement of multiple terminals: Part I”. IEEE Trans. Inf. Theory.

[24] Schneidman E., Still S., Berry M.J., Bialek W. (2003). Network information and connected correlations. Phys. Rev. Lett..

[25] Jaynes E.T., Jaynes E.T. (1983). Where do we stand on maximum entropy?. Essays on Probability, Statistics, and Statistical Physics.

[26] Amari S. (2001). Information geometry on hierarchy of probability distributions. IEEE Trans. Inf. Theory.

[27] McGurk H., MacDonald J. (1976). Hearing lips and seeing voices. Nature.

[28] Ince R.A.A. (2017). The partial entropy decomposition: Decomposing multivariate entropy and mutual information via pointwise common surprisal. arXiv.

